# Role of Routine Mid-Trimester Uterine Artery Doppler for Surveillance of Placental Mediated Disorders in a Low-Risk Population

**DOI:** 10.7759/cureus.30826

**Published:** 2022-10-29

**Authors:** Pooja Ramesh, Sudha Sumathy

**Affiliations:** 1 Department of Obstetrics and Gynaecology, Amrita Institute of Medical Sciences, Kochi, IND

**Keywords:** neonatal outcome, pre-eclampsia, placental insufficiency, fetal growth restriction, doppler in pregnancy

## Abstract

Objectives: Abnormalities in the placentation process can increase pregnancy-related complications like pre-eclampsia, placental abruption, intrauterine-fetal death (IUFD) or foetal-growth restriction (FGR). Our objective was to investigate the feasibility of utilising the mid-trimester uterine artery Doppler Pulsatility Index (PI), a non-invasive and effective screening tool, as a diagnostic measure to predict adverse pregnancy outcomes in a low-risk population in South India.

Materials and methods: This prospective cohort study was done in the Obstetrics and Gynaecology unit at Amrita Institute of Medical Sciences, South India, between August 2018 and January 2020. Uterine artery Doppler was performed along with the targeted anomaly scan between 18 and 24 weeks of gestation and a relationship was established with pregnancy outcome.

Results: Of 100 participants, abnormal uterine artery PI (PI > 90th centile) was found in 13 pregnancies, of which statistically significant association was found with hypertensive disorders (P=0.001), FGR (P=0.064) and preterm birth before 37 weeks (P=0.051). No association was found between abnormal uterine artery PI and neonatal birth weight (P=-0.3), APGAR score (P=0.35) and NICU admission (P=0.078).

Conclusion: An early abnormal finding in the doppler study can modify the level of antenatal surveillance required along with appropriate timely interventions, thereby significantly reducing the associated maternal and neonatal morbidity and mortality. When combined with routine ultrasound in pregnancy, such an affordable and straightforward diagnostic modality can improve antenatal care by reducing complications even in a low-risk population.

## Introduction

The ultimate purpose of good antenatal care is effective surveillance and timely intervention of high-risk pregnancies and their associated complications. The process of placentation is highly complex and begins early at the time of implantation. Hence, the perfect adaptation of vasculature between maternal and foetal circulation is vital. These adaptations aim to diminish the resistance in the vascular bed and thus increase the uteroplacental blood flow, improving the circulation to the foetus and ensuring optimum foetal development. Any abnormalities in the placentation process can increase the likelihood of pregnancy-related complications like pre-eclampsia, placental abruption, foetal growth restriction (FGR) and intrauterine foetal death (IUFD) [1].

It has been reported in a few studies that uterine artery Doppler is a valuable screening modality that can be utilised to recognise placental-mediated disorders in pregnancy. Doppler ultrasound examination is simple and accurately studies the state of uteroplacental circulation. As the gestation progresses, the impedance to flow within the uterine artery reduces during the course of a normal pregnancy. The failure in the physiological conversion of normal maternal placental arteries into low-resistance vessels leads to increased impedance to flow in the uterine arteries, resulting in pre-eclampsia or FGR [2].

Over the years, different Doppler techniques have been used for sampling. These include continuous-wave Doppler without visualising the vessel, pulsed-wave Doppler after identifying the vessel using B-mode ultrasound and colour-pulsed wave and colour flow imaging to identify the vessel, followed by pulsed-wave Doppler. The definition of abnormal flow velocity waveform used so far includes the Resistance Index (RI), Pulsatility Index (PI), and the presence of early diastolic notching. The Fetal Medicine Foundation (FMF), United Kingdom, states that the PI is currently the most popular Index for evaluating Doppler waveforms. Uterine artery PI, in particular, supplies us with an estimated measurement of the blood flow within the uterine and placental vasculature. An increased value can suggest some abnormality or defect in placentation with a consequent increased risk of developing pre-eclampsia, FGR, placental abruption and stillbirth [3].

Although mid-trimester uterine artery Doppler velocimetry is a useful screening tool in a high-risk population, its predictive value for diagnostic purposes is limited in the low-risk population. According to the latest WHO report, the world maternal mortality ratio was 211 deaths per 100,000 live births, while in India alone, it was estimated to be 113 per 100,000 live births. In other words, 94% of all maternal deaths were found to occur in low and lower-middle-income countries. Also, it was found that the third most important cause is hypertensive disorders in pregnancy, attributing to 10% of the total [4]. This certainly highlights the importance of early detection and management of these adverse pregnancy outcomes. The search for the single most potent screening test for early diagnosis of placental-mediated conditions has continued for several years. Newer screening modalities, such as the estimation of cell-free foetal DNA (cffDNA) and biomarkers like sFlt-1, placental growth factor, and placental protein 13, are not affordable and only challenging for the general public in low-resource settings to easily avail. Unfortunately, not many explorations of this kind have been executed in the southern part of the Asian continent and the existing studies published over the last few years have given us inconclusive results [5].

The main objective of this study was to understand the feasibility of using mid-trimester uterine artery Doppler in routine antenatal care in the general population by studying the link between abnormal mid-trimester uterine artery Doppler with unfavourable pregnancy outcomes. The maternal outcome measures mainly included hypertensive disorders, FGR, gestational age at delivery and spontaneous preterm birth and the neonatal outcomes were APGAR score at birth, birth weight and neonatal unit admissions.

This study was previously presented as a poster at the International Society of Ultrasound in Obstetrics and Gynaecology (ISUOG) meeting in October 2021.

## Materials and methods

This prospective cohort study was conducted at Amrita Institute of Medical Sciences, Kochi, South India, between the period, of August 2018 to January 2020. The study participants were selected at random from the antenatal clinic attendance and included Indian women between 20 and 35 years of age with an uncomplicated, low-risk and spontaneous singleton conception between 18 and 24 weeks of gestation. Pregnancies complicated by maternal illness, foetal/chromosomal abnormalities or those patients on anticoagulants were excluded from the study.

The study design was approved by the institution's Research Ethics committee (Approval number- IEC-AIMS-2017-OBS.GYNEC-377). Maternal demographic information and relevant antenatal details were obtained from the hospital's electronic medical records. The gestational age was assumed based on the last menstrual period. However, if there was a discrepancy in dates of ≥ 7 days, an ultrasound scan between 11 and 13 weeks gestation was used to assign a due date. If the date of the last menstrual period was unreliable and the 11-13 weeks scan was unavailable, the second-trimester scan was used to reassign the age. 

Uterine artery Doppler is an additional investigation that is performed in our institute, along with the routine anomaly scan in the second trimester. These patients were then actively followed up until delivery and a relationship was established between uterine artery Doppler patterns (PI) and pregnancy outcome.

Uterine artery Doppler

**Figure 1:**
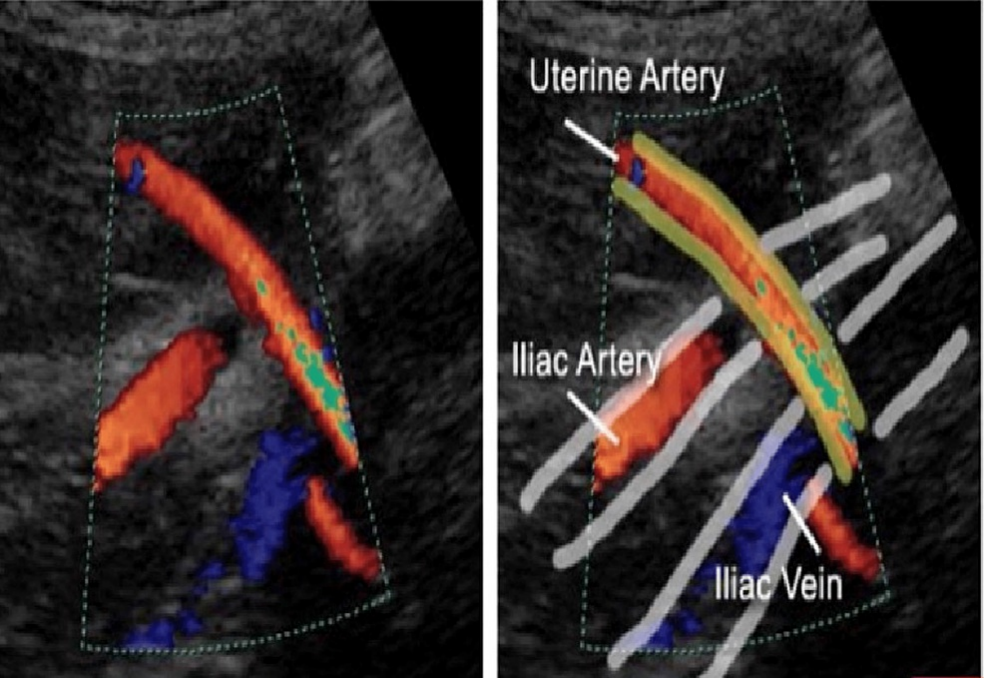
Site of insonation of uterine artery (“crossing over”)

In foetal medicine, the Doppler principle is used to evaluate any changes in the sound waves that denote the direction and velocity of blood flowing through the vessels and heart. This information can be plotted against time to understand the features of maternal and foetal blood circulation. The uterine arteries form the primary source of blood supply to the uterine muscles and placenta. The two arteries on the left and right sides of the uterus are located at the site of crossing over the iliac vessels. The evaluation can be done by direct visualisation by analysing the various attributes of the Doppler waveforms in order to demarcate the presence of notching (Figure 1). One may also quantify the velocity of the blood flow, both at peak systole and diastole. While the peak systole corresponds to the contraction of the heart, the peak diastole is linked to its relaxation. These measures are then tallied to emanate a ratio. One of the standard practices considered in several past studies is to measure the RI, in which the peak of systole is divided by the sum of systole and diastole. After the Doppler values are computed, the outcomes are graphically represented to determine if there is any aberration in the blood flow during diastole. According to FMF, the PI is regarded to be increased if it is beyond the 90th centile for that particular period of gestation [6]. This has been used as the cut-off for the purpose of this study.

Statistical analysis

IBM SPSS version 20.0 software (IBM Corp., Armonk, NY) was used for statistical analysis. While the categorical variables are presented as frequency and percentage, the continuous variables are expressed as mean and standard deviation. The Chi-square test is used to determine the statistical significance of the relationship between abnormal uterine artery Doppler and pregnancy outcomes. A p-value less than 0.05 was considered to determine statistical significance. Wherever appropriate, diagnostic measures like sensitivity (Sn), specificity (Sp), positive predictive value (PPV) and negative predictive value (NPV) were also calculated.

## Results

**Figure 2:**
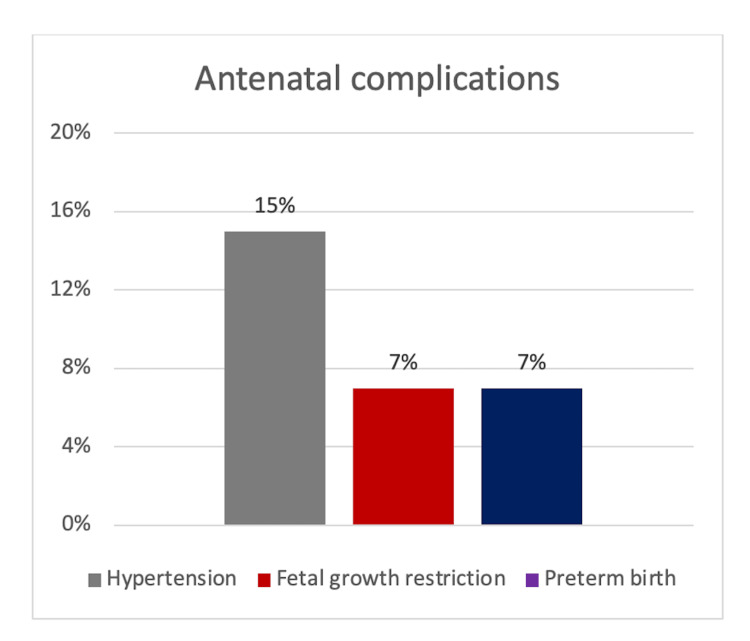
Graphical illustration of the antenatal complications

In this study of 100 participants, the mean age was found to be 27.4 years (SD=4.7) and 41% (n=41) were nulliparous women. It was found that 15% (n=15) of the sample developed hypertensive disorders, while 7% (n=7) had FGR and 7% (n=7) had spontaneous preterm birth before 37 weeks of gestation (Figure 2). All these complications were noted to have occurred in the third trimester of the pregnancy.

A raised value of uterine artery Doppler PI of more than the 90th centile was seen in 13 of the total pregnancies and 69.2% (n=9) of these pregnancies were complicated by Hypertension during the ante-natal period (after 20 weeks). This association was statistically significant with a p-value of 0.001 with a Sn of 60% and a Sp of 95.3%. The PPV and NPV were 69% and 93.1%, respectively (Table 1). Further, 23.1% (n=3) of pregnancies with raised PI were found to have FGR (p-value=0.064) with a Sn of 44.4%, Sp of 90%, PPV of 30.8% and NPV of 94.9% (Table 1). These participants were managed appropriately with anti-hypertensives and surveillance except only one of them who developed severe pre-eclampsia with associated FGR at 34 weeks that required the pregnancy to be terminated.

**Table 1:** Diagnostic measures denoting the diagnostic value of mid-trimester uterine artery Doppler to predict adverse pregnancy outcomes in this study

Variables	Sensitivity (Sn) (%)	Specificity (Sp) (%)	Positive Predictive Value (PPV) (%)	Negative Predictive Value (NPV) (%)
Hypertensive disorders	60	95.3	69.2	93.1
Foetal growth restriction	44.4	90	30.8	94.3

The mean gestational age at the time of delivery was found to be 37 weeks 5 days (SD=2 weeks 3 days). Of the 100 patients, 80% (n=80) of them had a term delivery (>37 weeks), while 16% (n=16) delivered between 32-37 weeks and 4% (n=4) between 27-32 weeks. Of the preterm births, 35% (n=7) had a spontaneous preterm birth, while the remaining pregnancies were terminated before 37 weeks in view of adverse maternal or foetal outcomes secondary to placental insufficiency. Abnormal uterine artery PI was found to be linked to 38.5% (n=5) preterm birth between 32 and 36 weeks 6 days, giving a statistically significant result with a p-value of 0.051. However, no direct relationship was noted with spontaneous preterm labour (p-value=1.00).

Out of the 100 neonates delivered, 6% (n=6) had an APGAR score of <7, taken at the time of delivery. 17% (n=17) of the neonates weighed below 2.5 kg and 12% (n=12) mandated neonatal special care admission following birth for varied reasons. No connection was seen between raised uterine artery PI and neonatal birth weight (p-value=0.3), low APGAR score at birth (p-value=0.35) and neonatal special care unit admission (p-value=0.078) (Table 2).

**Table 2:** Statistically significant outcome results P-value less than 0.05 was considered to determine statistical significance

Outcome measures	Uterine artery Doppler Pulsatility Index	Yes, n (%)	No, n (%)	P-value
Hypertensive disorders	>/= 90th centile (13)	9 (69.2)	4 (30.8)	0.001
	< 90th centile (87)	6 (6.9)	81 (93.1)	
Foetal growth restriction	>/= 90th centile (13)	3 (23.1)	10 (76.9)	0.064
	< 90th centile (87)	4 (4.6)	83 (95.4)	
Preterm Birth ( < 37 weeks)	>/= 90th centile (13)	5 (38.5)	8 (61.5)	0.051
	< 90th centile (87)	15 (17.2)	72(82.8)	

## Discussion

Over the last decade, screening uterine artery Doppler has evolved from a blind method (continuous wave Doppler) to localising vessels using colour flow mapping. This significantly diminished the difficulty in distinguishing uterine artery blood flow from the high-resistance internal iliac vessels and low-resistance arcuate arteries located in close proximity to each other. Screening using uterine artery Doppler has become quite popular in day-to-day clinical practice due to its effectiveness and affordability. However, the reality is that, currently, the only cure for pre-eclampsia and related diseases is the termination of pregnancy and although diagnosing these disorders early does not mean an early cure, it can undoubtedly help clinicians be more vigilant and prevent or treat maternal as well as foetal complications earlier [7]. The lack of a timely diagnosis and recognition of known risk factors results in substandard outcomes. Studies have proven that specific measures taken accordingly, such as the early administration of prophylactic aspirin before 16 weeks of gestation in women at risk of developing uteroplacental insufficiency, can lower the chance of developing pre-eclampsia by 17%, preterm birth by 8% and 14% reduction in foetal and neonatal mortality [8].

This study intended to assess if mid-trimester uterine artery Doppler velocimetry had any difference in predicting pregnancy outcomes in a low-risk population. As expected, those women with normal impedance to flow in the uterine artery had a low risk of developing uteroplacental insufficiency. Similar to a few other studies demonstrating a favourable correlation with second-trimester Doppler measures of the uterine artery, this study ascertained to be beneficial in anticipating the association of elevated uterine artery PI with the development of hypertensive disorders, FGR and preterm birth in pregnancy with good specificity, mainly when applied to women at high risk or with a previous history of similar complaints (Table 3).

**Table 3:** Studies that proved the association of second-trimester uterine artery Doppler with pregnancy outcome FGR - Foetal growth restriction, SGA - Small for gestational age, LBW - Low birth weight, IUD - Intrauterine death, LSCS - Lower segment Caesarean section

S.No	Study	Year	Uterine artery Doppler	Outcome
1.	Dhar et al. [9]	2017	Abnormal PI- ≥95^th^ percentile	Significant association was noted with pre-eclampsia, FGR and LBW. No association with preterm labour, IUD or abruption
2.	Barati et al. [10]	2014	Abnormal PI- mean PI >1.45	Significant association was noted with Hypertensive disorders and SGA.
3.	Verma et al. [11]	2016	Abnormal uterine artery doppler- Bilateral uterine artery notches or mean PI ≥1.45 ( >95^th^ percentile)	Significant association was noted with pre-eclampsia, FGR, LBW and preterm delivery.
4.	Scandiuzzi et al.[12]	2016	Abnormal Uterine artery RI and PI> 95^th^ centile.	Positive association with hypertensive disorders. No association with hypertensive disorders, SGA, Mode of Delivery (LSCS rate), APGAR score at birth and neonatal special care admission.

In line with the findings of the current study, Dhar et al. conveyed meaningful outcomes in their study conducted in 2017, where elevated uterine artery PI, which was taken to be ≥95th percentile, had a clear association with pre-eclampsia and FGR (p-value=0.001) with an Sn of 35.7% and Sp of 98.2%. However, no association was found with spontaneous preterm labour, IUD and abruption [9]. Again, another study by Barati et al. in 2012 also proved that abnormal uterine artery Doppler PI helped predict only hypertensive disorders and FGR but with good sensitivity and specificity of 79% and 95.5%, respectively, at a 95% confidence interval [10].

In 2016, Verma et al. considered uterine artery PI and bilateral uterine artery notching and proved the association of abnormal Doppler with pre-eclampsia and FGR. However, no association was found in the present study with neonatal birth weight, unlike in their study (p-value=0.001) [11]. As opposed to this study, a more extensive study by Scandiuzzi et al. in 2016 included uterine artery Doppler screening more than once in pregnancy but found a significant positive association between abnormal uterine artery RI and PI>95th centile and hypertensive disorders and neonatal special care admission only in the first trimester. No association was found with hypertensive disorders, small for gestational age (SGA), Mode of Delivery, APGAR score at birth and neonatal unit admission in later stages of pregnancy (Table 3) [12].

Limitations 

Though the prospective design is the biggest strength of the study, both the sample size and the total participants with elevated uterine artery PI values were trivial in relation to the large population and this could have led to the statistical insignificance of the results of certain outcome measures. Therefore, we require extensive studies in this area with a good sample size and adequate randomisation.

Also, in this study, only the PI was used as an indicator of uteroplacental blood flow, unlike most other analyses in the past, where other measures like RI and diastolic notching were also regarded to demonstrate abnormal Doppler waveforms. These other units have undoubtedly enhanced the diagnostic value and accuracy in predicting placental-mediated disorders [12,13].

## Conclusions

In conclusion, this study proved the usefulness of uterine artery doppler as a highly effective screening method for predicting adverse foetal and maternal outcomes in a low-risk population. Even though there are no effective interventions currently available to treat or reverse the course of the disease in these women, starting prophylactic Aspirin prior to 16 weeks of gestation after the recognition of risk factors and an early diagnosis of abnormal doppler parameters can be used to judiciously modify the level of antenatal surveillance and management in low resource settings, thereby significantly reducing the associated maternal and neonatal morbidity and mortality.

Due to the multitude of pathogenic aspects like genetic and ethnic variations, it is essential to perform a comprehensive analysis until a reasonable consensus may be attained. This study has given us promising results with potential clinical implications, which adds to the expanding body of research and helps in the early recognition and appropriate vigilance of placental-mediated disturbances in pregnancy. When combined with routine ultrasound in pregnancy, such an affordable and straightforward diagnostic modality can broaden the prospects for more effective antenatal care and management, ultimately reducing such undesired complications.
